# Supplementary material for: The Effects of Epicuticular Wax on Anthracnose Resistance of Sorghum bicolor
Source: Int J Mol Sci. 2023 Feb 4;24(4):3070. doi: 10.3390/ijms24043070 (PMC9964091; doi:10.3390/ijms24043070)
Supplement: Supplementary file 1 [file ijms-24-03070-s001.zip › Supplementary figures and tables.pdf]

Supplementary figures and tables

Table S1 Genes differentially expressed in sorghum leaves remaining epicuticular wax (a) and removed of epicuticular wax (b) after anthracnose infection at 3 dpi.

Table S2 DEGs annotated to the KEGG pathway.

Table S3 Calculation of disease grades and disease index in sorghum.

Table S4 Total epicuticular wax amounts and their individual compounds amounts of leaves sampled from anthracnose infection sorghum in field.

Table S5 Primers used in this study.

Table S6 Classification and evaluation criteria of resistance to anthracnose in sorghum.

Table S7 The amounts of wax component in sorghum leaves.

Table S8 The KI and m/z of identified wax components using GC-MS

Figure S1 Isolation and identification of anthracnose *Colletotrichum sublineola* from sorghum in the field.

Figure S2 Venn diagram showing up-regulated (a) and down-regulated (b) DEGs in sorghum leaves remaining epicuticular wax and removed of epicuticular wax after anthracnose infection at 3 dpi.

Figure S3 Log_2fold values of qRT-PCR data.

Table S3 Calculation of disease grades and disease index in sorghum.

Cultivars	Disease grades					Disease index	Resistance evaluation
	1	2	3	4	5		
705013	40	10	0	0	0	20	HR
Baizhan	41	5	3	0	1	8	HR
Hupo	41	10	2	1	1	10	HR
Minge	30	16	2	0	0	21	R
Mule8000	28	8	8	0	0	27	R
Haishi	27	12	3	1	1	14	R
African giant	20	16	4	0	0	30	R
E35	32	8	4	0	4	17	R
Kangdi	25	18	5	3	1	20	R
Dalishi	35	10	6	4	4	21	MR
Yajin No.3	19	10	2	1	5	25	MR
Jiajiang	22	10	4	3	4	25	MR
Pengshui	27	10	4	6	4	25	MR
Pin 05206	21	18	2	3	5	26	MR
Feng FH59	27	12	0	6	6	26	MR
Luxian	25	12	5	4	5	26	MR
Chaobei-Huangfan-2	26	11	3	1	8	27	MR
Chuannuoliang No. 2	42	13	7	0	15	28	MR
Luzhou	16	10	8	2	4	30	MR
Jiyuliang No.3	19	12	5	3	7	32	MR
Jinnuoliang No.1	23	17	6	2	10	32	MR
sug High stem	46	8	4	8	17	33	MR
Shanxi (H1)	20	20	11	7	7	35	MR
Hongyingzi	18	20	6	6	8	35	MR
Jintian No.1	21	12	1	5	11	37	MR
GW0730	18	15	3	6	9	37	MR
BJ0603	28	2	6	2	19	42	S
Xinjin	25	7	2	2	19	42	S
G12551	9	15	15	9	6	44	S

Table S4 Total epicuticular wax amounts and their individual compounds amounts of leaves sampled from anthracnose infection sorghum in field.

**Cultivars**	**Total wax content** (μg/cm^2)	**Alkanes** (μg/cm^2)	**Aldehydes** (μg/cm^2)	**Primary alcohols** (μg/cm^2)	**Alkanoic acids** (μg/cm^2)
705013	15.34	3.08	3.07	3.91	0.72
Baizhan	15.28	2.72	3.87	2.93	1.06
Hupo	13.13	2.51	3.90	2.04	1.22
Minge	16.35	2.86	4.66	3.78	1.30
Mule8000	12.58	2.73	2.71	3.30	0.72
Haishi	13.00	3.58	3.43	1.67	1.01
African giant	10.82	2.27	2.36	3.19	0.59
E35	12.29	2.53	2.82	2.98	0.74
Kangdi	14.10	2.76	3.93	2.78	1.18
Dalishi	13.04	2.25	2.94	2.80	0.86
Yajin No.3	10.50	2.31	2.47	1.48	0.68
Jiajiang	12.92	2.85	3.00	3.02	1.00
Pengshui	9.09	2.00	3.37	0.92	0.99
Pin 05206	8.49	2.56	3.54	1.52	0.87
Feng FH59	8.59	3.01	3.15	1.58	0.85
Luxian	10.43	2.03	2.53	3.16	0.77
Chaobei-Huangfan-2	10.19	2.06	2.14	2.07	0.64
Chuannuoliang No. 2	8.01	2.00	2.06	1.37	0.58
Luzhou	12.40	3.11	3.36	1.69	1.13
Jiyuliang No.3	8.00	1.33	1.41	1.04	0.33
Jinnuoliang No.1	9.58	2.31	2.30	2.13	0.68
sug	9.14	2.77	1.82	2.16	0.48
Shanxi (H1)	5.10	1.25	1.39	1.12	0.45
Hongyingzi	12.13	3.07	2.91	2.75	0.80
Jintian No.1	11.15	2.09	3.25	2.61	1.03
GW0730	7.20	2.34	2.98	2.52	0.90
BJ0603	9.05	2.22	2.93	1.36	0.70
Xinjin	7.22	1.58	2.75	0.93	0.77
G12551	10.88	2.23	3.26	1.98	0.96

Table S5 Primers used in the study.

Gene	Gene ID	Primer sequences (forward)	Primer sequences (reverse)
SbKAS11	Sobic.004G086800	TCAACTGGTACTCGGGCAAC	AGCGTCGTGATGTTGGTCTT
SbACC1	Sobic.008G114300	GAGGACTGCGAAAGGGAATG	GGCATCAACTGCTTCATCCG
SbKCS5	Sobic.004G249400	CCTACATCGAGGCCAAGGGAC	CTTGAGCACGTCGGGAATGT
SbKCS17	Sobic.009G244300	GTTTACGACAAGGACACGCC	TCCAGCAGGTCGTAGTAGCG
SbActin	Sobic.001G112600	TGGCATCCCTCAGCACCTTCC	AATGGCTCCTCTCGGCTTGC

Table S6 Classification and evaluation criteria of resistance to anthracnose in sorghum.

Disease grade	Representative value	Lesion area and description	Resistance evaluation
1	0	No obvious symptoms	Highly resistant (HR)
2	1	The area of symptom-free lesion accounted for 1%-10% of the leaf area, but no conidia disk was produced	Resistant (R)
3	2	The area of lesion accounted for 11%-25% of the leaf area, but no conidia disk was produced	Middle resistant (MR)
4	3	The area of lesion without conidia accounted for 26%-50% of the leaf area, and conidia disk was produced	Susceptible (S)
5	4	The lesion area of conidia disk was more than 50% of the leaf area, and conidia disk was produced	Highly susceptible (HS)

Table S7 The amounts of wax component in sorghum leaves.

wax component	Amount (×10^{-2} μg/cm^2)			
	+EW_CK	+EW_T	-EW_CK	-EW_T
Heptacosanal	0.21±0.030	0.29±0.006	0.16±0.016	0.13±0.024
Octacosanal	3.68±0.037	1.56±0.108	2.12±0.027	2.10±0.203
Nonacosanal	0.09±0.013	0.06±0.007	0.07±0.007	0.04±0.012
Triacontanal	5.65±0.095	4.12±0.079	4.70±0.043	2.09±0.283
Hentriacontanal	0.32±0.032	0.13±0.009	0.18±0.010	0.17±0.038
Dotriacontanal	12.49±1.337	10.12±0.879	6.65±0.054	5.41±0.338
Tetratriacontanal	0.46±0.003	0.39±0.045	0.32±0.025	0.23±0.127
n-Pentacosane	0.57±0.015	0.24±0.000	0.26±0.009	0.66±0.205
n-Hexacosane	0.58±0.020	0.19±0.006	0.34±0.002	0.43±0.098
n-Heptacosane	1.28±0.026	0.86±0.065	0.73±0.014	1.01±0.154
n-Octacosane	1.21±0.046	0.53±0.016	0.92±0.101	1.17±0.042
n-Nonacosane	3.47±1.600	5.29±0.267	3.72±0.090	4.17±0.093
n-Triacontane	0.61±0.022	0.31±0.005	0.28±0.001	0.43±0.027
n-Hentriacontane	5.00±0.085	3.64±0.037	2.36±0.061	2.48±0.068
n-Tritriacontane	0.98±0.036	1.04±0.019	0.62±0.040	0.55±0.040
n-Pentatriacontane	0.44±0.045	0.13±0.005	0.31±0.004	0.27±0.054
Arachidic acid	0.22±0.017	0.07±0.003	0.04±0.008	0.29±0.047
Docosanoic acid	0.28±0.046	0.17±0.015	0.13±0.012	0.21±0.064
Lignoceric acid	3.27±1.150	0.50±0.040	0.42±0.033	0.60±0.034
Cerotic acid	1.00±0.014	0.12±0.007	0.20±0.029	0.72±0.037
Montanic acid	1.54±0.002	0.50±0.016	0.52±0.046	1.32±0.104
Melissic acid	3.98±0.317	2.53±0.194	1.77±0.009	1.54±0.074
Lacceroic acid	0.47±0.063	0.26±0.007	0.18±0.004	0.25±0.025
n-Hexacosanol	0.25±0.061	0.34±0.015	0.18±0.007	0.15±0.033
n-Octacosanol	4.73±0.141	3.01±0.187	1.99±0.107	0.42±0.066
n-Triacontanol	4.04±0.096	3.01±0.050	2.11±0.026	1.96±0.529
n-Tridodecanol	0.73±0.166	0.16±0.005	0.31±0.050	0.50±0.130
a-amyrin	0.81±0.150	0.90±0.079	0.59±0.050	0.59±0.052
β-amyrin	1.25±0.040	0.40±0.000	0.68±0.055	1.42±0.464

Table S8 The KI and m/z of identified wax components using GC-MS

Wax component	KI	m/z
Heptacosanal	3135	376/394
Octacosanal	3274	390/408
Nonacosanal	3428	404/422
Triacontanal	3624	418/436
Hentriacontanal	3747	432/450
Dotriacontanal	3917	446/464
Tetratriacontanal	4076	474/492
n-Pentacosane	2494	352
n-Hexacosane	2610	366
n-Heptacosane	2720	380
n-Octacosane	2849	394
n-Nonacosane	3015	408
n-Triacontane	3191	422
n-Hentriacontane	3379	436
n-Tritriacontane	3709	464
n-Pentatriacontane	4022	492
Arachidic acid	2440	369/384
Docosanoic acid	2647	397/412
Lignoceric acid	2919	425/440
Cerotic acid	3307	453/468
Montanic acid	3648	481/496
Melissic acid	3893	509/524
Lacceroic acid	4151	537/552
n-Hexacosanol	3098	440/455
n-Octacosanol	3461	468/483
n-Triacontanol	3779	496/511
n-Tridodecanol	4043	524/539
a-amyrin	3831	426
β-amyrin	3731	426

For aldehyde, the m/z refers to M-18/M+; for alcohol and acids (TMS-derivative), the m/z refers to M-15/M+. The KI was calculated with the following equations: RI = $100*z+100*(RT_x-RT_z)/(RT_{z+1}-RT_z)$, where RT_x refers to the retention time of tested compound; RT_z refers to the retention time of n-alkane with carbon number n; RT_{z+1} refers to the retention time of alkane with carbon number n+1.

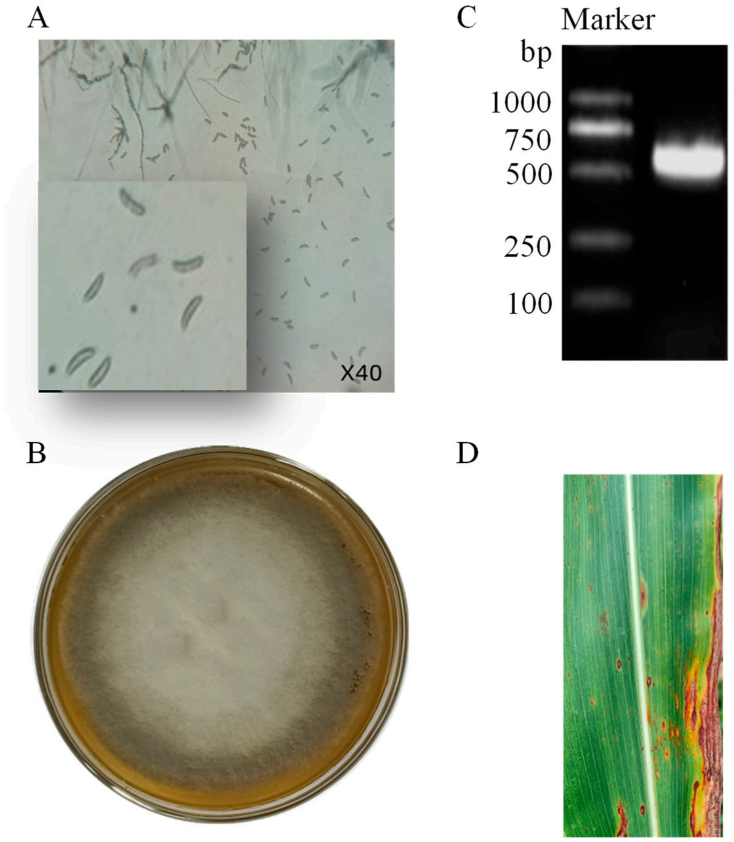

Figure S1 Isolation and identification of anthracnose *Colletotrichum sublineola* from sorghum in the field. A-B, The colony (A), conidia (B) morphological characteristics of *C. sublineola*. C, The amplified sequence of *C. sublineola* amplified with primer ITS1/4. D, Disease lesions generated from inoculation of the isolated *C. sublineola* strain on sorghum for 7 days.

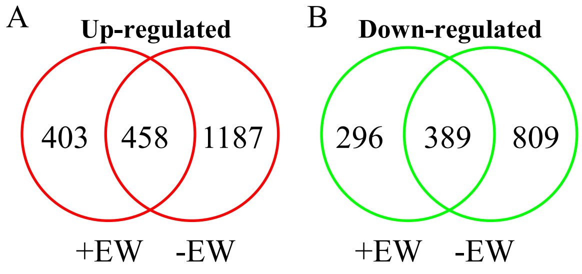

Figure S2 Venn diagram showing down-regulated (a) and up-regulated (b) DEGs in sorghum leaves remaining epicuticular wax and removed of epicuticular wax after anthracnose infection at 3 dpi.

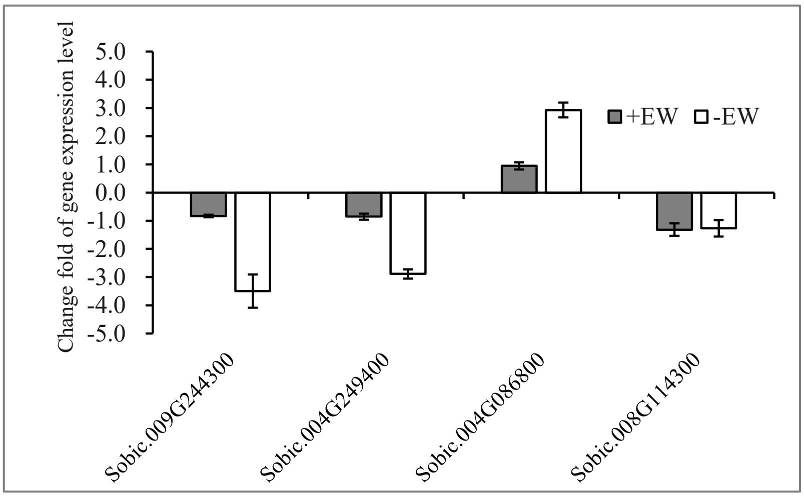

Figure S3 Log_2fold values of qRT-PCR data.
